# The associations between tobacco exposure and diarrhea within the National Health and Nutrition Examination Survey (NHANES)

**DOI:** 10.18332/tid/224313

**Published:** 2026-08-07

**Authors:** Jiaheng Shao, Chuxuan Huang, Xilin Chen, Houyu Zhao

**Affiliations:** 1 Foshan Clinical Medical School of Guangzhou University of Chinese Medicine, Foshan, China; 2 Foshan Fosun Chancheng Hospital, Foshan, China; 3 The First Clinical Medical School of Guangzhou University of Chinese Medicine, Guangzhou, China; 4 Department of Oncology, The First Affiliated Hospital of Xinxiang Medical University, Weihui, China

**Keywords:** diarrhea, tobacco exposure, secondhand smoke, epidemiology

## Abstract

**INTRODUCTION:**

As a common clinical symptom, diarrhea imposes considerable burdens on patients' lives and healthcare. Smoking and secondhand smoke (SHS) exposure, as modifiable risk factors, currently exhibit an uncertain relationship with diarrhea, lacking relative studies to clarify the relationship. The study aims to clarify the relationship between different tobacco exposure situations and diarrhea.

**METHODS:**

This is a secondary analysis of 2005–2010 NHANES data, following a cross-sectional analytical design. Tobacco exposure in the population was divided into four groups (smokers, ex-smokers, never smokers exposed to SHS, and never smokers not exposed to SHS) based on smoking status and household SHS exposure. Diarrhea was ascertained using the Bristol Stool Form Scale (BSFS). Logistic regression analyses evaluated associations between tobacco exposure and diarrhea, with stratification analysis conducted to validate the robustness of findings. Additionally, we examined the potential mediating role of serum nicotine in this relationship.

**RESULTS:**

Compared to never smokers not exposed to SHS, smokers demonstrated a significantly elevated likelihood of diarrhea (AOR=1.56; 95% CI: 1.25–1.93). However, this association was not observed among ex-smokers or never smokers exposed to SHS. Among smokers, those who reported smoking immediately after waking showed an increased likelihood of reporting diarrhea (AOR=1.69; 95% CI: 1.17–2.45). Each additional year of smoking duration was associated with a 2% increase in the likelihood of diarrhea (AOR=1.02; 95% CI: 1.00–1.03) while daily cigarette consumption showed no significant association. Serum cotinine was not a mediator in the relationship between tobacco exposure and diarrhea.

**CONCLUSIONS:**

Active tobacco exposure is associated with an elevated likelihood of diarrhea, whereas no association was found for either passive smoking or former smoking. Both smoking duration and smoking immediately upon waking exhibit positive associations with heightened diarrhea likelihood, suggesting potential intestinal health benefits from smoking cessation at an earlier stage. Serum cotinine did not mediate this relationship.

## INTRODUCTION

Diarrhea is a common gastrointestinal disorder clinically defined by loose stools and more frequent bowel movements^1^. Despite significant global efforts in improving hygiene conditions and public health awareness, diarrhea remains one of the significant contributors to the global disease burden^2^. Previous research^3^ has shown that chronic diarrhea or loose or watery stools affected up to 26.9% of adults in the United States, severely impacting people’s quality of life. Furthermore, according to data from 2016, there were over 4.4 billion cases of diarrhea worldwide, resulting in 1.6 million deaths^2^, generating substantial medical and healthcare costs, and having a profound impact on socioeconomy^4^. Additionally, chronic diarrhea is positively associated with the risk of colon cancer, cardiovascular diseases, and all-cause mortality^5^.

Smoking, a modifiable risk factor, has long been recognized as closely linked to respiratory and cardiovascular diseases^6,7^. However, its impact on the gastrointestinal tract remains a complex area of research. Previous studies have indicated that smoking is a risk factor for Crohn’s disease (CD) and irritable bowel syndrome (IBS)^8,9^, while other studies have noted conflicting results^10,11^. Although the aforementioned conditions may present with diarrhea symptoms^12^, research directly investigating the association between tobacco exposure and diarrhea remains scarce to date. Meanwhile, the relationship between exposure to SHS and bowel health also remains controversial^13-15^. Therefore, further research is required to clarify the relationship between smoking and diarrhea, rather than merely within the framework of disease specificity. Given this background, we utilized the available data from NHANES to assess the relationship between different tobacco exposure situations and diarrhea.

## METHODS

### Study population and data sources

This is a secondary dataset analysis of data from the National Health and Nutrition Examination Survey (NHANES), which represents a continuous cross-sectional surveillance program employing a stratified, multistage probability-clustering sampling strategy to obtain nationally representative data from non-institutionalized United States civilians^16^. This surveillance system, administered by the National Center for Health Statistics (NCHS), operates under Protocol 2005–2006 with formal authorization from the NCHS Institutional Review Board. Written informed consent was obtained from all participants following ethical review and approval by the National Health Statistics Ethics Review Committee (Protocol No. 2005-06).

Our study participants were derived from NHANES conducted between 2005 and 2010, for which we have pooled the data of three waves of investigation: 2005–2006, 2007–2008, 2009–2010, for a cross-sectional approach. Participants were included in the study if they met the following criteria: aged ≥20 years, had complete tobacco exposure information, completed the Gut Health Questionnaire, and had complete covariate information.

### Bowel health questionnaire

The Bowel health questionnaire was completed in the Mobile Examination Center (MEC) survey room using a computerized personal survey system. The BSFS, commonly used in research and clinical practice, was used to define chronic diarrhea. Participants were shown a card with color pictures and descriptions of the seven BSFS (types 1 to 7)^17^. Participants who indicated their typical or most frequent stool type as BSFS Type 6 or BSFS Type 7 were classified as having diarrhea. Other participants were classified as having normal bowel habits^18^.

### Exposure: Smoking

The tobacco exposure situations were evaluated by trained interviewers using the Computer-Assisted Personal Interviewing (CAPI) system. Based on the classification of smoking status, this study further categorizes participants’ exposure to SHS. Specifically, based on responses to the questions ‘Have you smoked at least 100 cigarettes in your lifetime?’ and ‘Do you currently smoke cigarettes?’, participants who answered ‘yes’ to both questions are considered ‘smokers’, those who answered ‘yes’ to the first question but ‘no’ to the second are considered ‘ex-smokers’, and those who answered ‘no’ to both questions are considered ‘never smokers’. For never smokers, based on their response to the question ‘Does anyone smoke inside your home?’, those who answered ‘yes’ are considered ‘never smokers exposed to SHS’, and those who answered ‘no’ are considered ‘never smokers not exposed to SHS’.

For smokers, the smoking duration is calculated as current age minus age at smoking initiation (age at smoking initiation was based on their answers to ‘Age started smoking cigarettes regularly?’). The average number of cigarettes consumed daily is calculated by their answers to ‘Avg cigarettes/day during past 30 days’. Based on the participants’ responses, they were categorized into three groups: 1–10, 11–20, and >20 cigarettes per day. Additionally, participants were classified as those smoking immediately after waking (within 5 minutes) or non-immediate smokers based on their answers to ‘How soon after waking do you smoke?’.

### Measurement of serum cotinine levels

Isotope dilution-high-performance liquid chromatography/atmospheric pressure chemical ionization tandem mass spectrometry was used to measure serum cotinine levels^19^. As suggested by a previous study^20^, we created cotinine categories using the newly recommended cutoff point of 3 ng/mL to represent smoking exposure^19^. Those below the lower detection threshold were considered unexposed.

### Covariates

Based on previous studies^21,22^ and our clinical experience, we preliminarily selected a set of potential factors as covariates to analyze the relationship between tobacco exposure and diarrhea, including gender, age, race (Mexican American, Other Hispanic, Non-Hispanic White, Non-Hispanic Black, and Other), education level (lower than high school, high school, higher than high school), marital status (married, unmarried, other), poverty-to-income ratio (PIR: <1.30, 1.30–3.49, ≥3.50)^21^, BMI (kg/m^2^) (normal weight <25, overweight 25–29.9, obese ≥30)^21^ , hypertension (hypertension, non-hypertension)^22^ and diabetes(diabetes, non-diabetes, borderline)^22^. The BMI data were measured in the MEC by trained health technicians. Other covariates were collected by trained interviewers using the CAPI system.

### Statistical analysis

All analyses accounted for sample weights. In descriptive analyses, categorical variables were presented through weighted percentages, whereas continuous variables were represented by means and standard deviations (SD). To detect variations in baseline characteristics between diarrhea and non-diarrhea patients for categorical data, the chi-squared test was used. For continuous variables, the t-test was applied to evaluate differences between groups.

Weighted logistic regression models were used to investigate the relationship between different tobacco exposure scenarios and diarrhea. First, with never smokers not exposed to SHS as the reference group, we evaluated the likelihood of having diarrhea among three groups: smokers, ex-smokers, and never smokers exposed to SHS. Additionally, for smokers (not including ex-smokers, never smokers not exposed to SHS, and never smokers exposed to SHS), using three separate regression models, we separately examined the effects of smoking time (immediate smokers and non-immediate smokers), smoking duration, and smoking dose on the likelihood of having diarrhea. For all the logistic regressions, we used three models. Model 1 did not account for any potential covariates. Model 2 was adjusted for age, sex, and race. Model 3 was additionally adjusted for marital status, education level, household income, BMI, hypertension, and diabetes. For smokers (not included ex-smokers, never smokers not exposed to SHS, never smokers exposed to SHS), restricted cubic spline (RCS) analysis with four knots (the 5th, 35th, 65th, and 95th quantiles), controlling for all covariates, was conducted to examine potential nonlinear relationships between smoking duration and the likelihood of diarrhea, and likelihood ratio tests were employed to test for nonlinear relationship. Stratification analyses were used to assess the robustness of the tobacco exposure-diarrhea association and to assess whether the association was modified by specific variables. The variables examined included gender, education level (lower than high school, high school, higher than high school), PIR (<1.30, 1.30–3.49, ≥3.50), BMI (normal weight, overweight, obese), hypertension (hypertension, and non-hypertension), and diabetes (diabetes, non-diabetes, and borderline). Except for the stratification component itself, all covariates were adjusted: age, gender, race, education level, marital status, PIR, BMI, hypertension, and diabetes.

The Baron and Kenny four-step method was employed to examine the mediating role of serum cotinine in the association between tobacco exposure and diarrhea: initially, the association between tobacco exposure (i.e. smokers) and diarrhea was confirmed based on the aforementioned analysis; subsequently, logistic regression analyses were used to investigate the associations between tobacco exposure and serum cotinine concentration; next, the association between serum cotinine concentration and chronic diarrhea was assessed and finally, serum cotinine concentration was included as a covariate to examine whether it could attenuate the association between tobacco exposure and diarrhea. Furthermore, for all multivariable models that may contain multicollinearity in this research, variance inflation factor (VIF) values were calculated to assess multicollinearity, with VIF <10 as the acceptable criterion.

All analyses were performed using R version 3.4.3, Empower software (X&Y Solutions, Inc., Boston, MA), and Stata software version 18 (StataCorp, College Station, TX, USA). A two-sided p<0.05 was considered statistically significant.

## RESULTS

### Basic characteristics of participants

**Figure 1:**
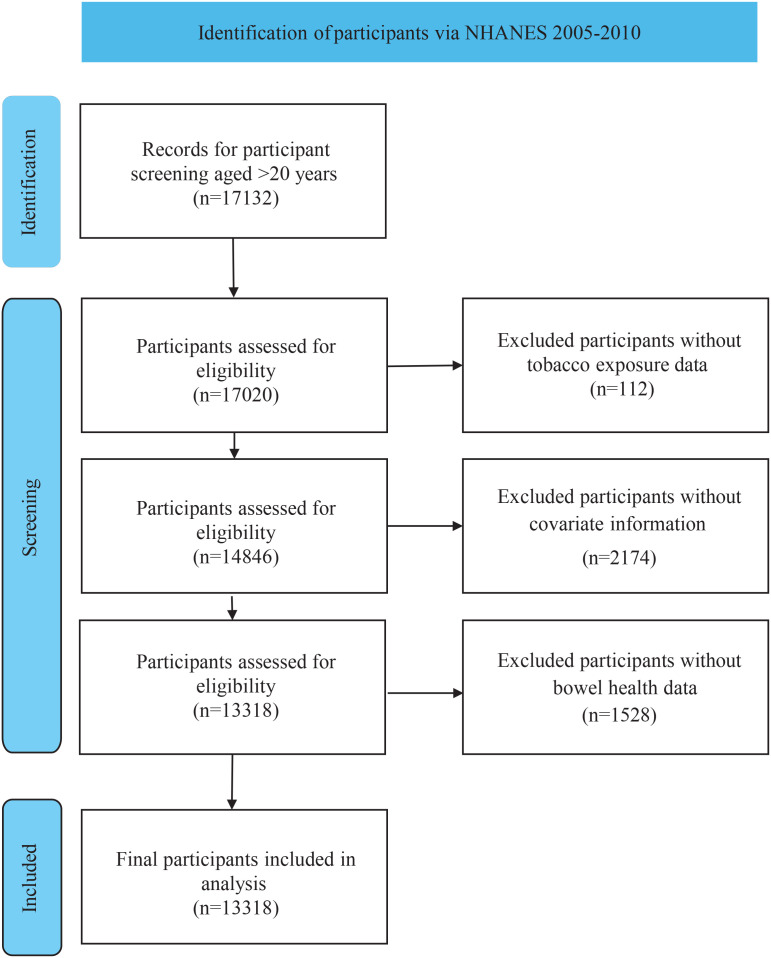
The screening process flowchart for the cross-sectional analysis of tobacco exposure and diarrhea in the National Health and Nutrition Examination Survey (NHANES) 2005–2010 (N=13318)

Participant selection followed a sequential exclusion process: Firstly, individuals without data on tobacco exposure were excluded (n=112). Then, participants lacking covariate information (n=2174) and those lacking data on bowel health (n=1528) were removed. Finally, 13318 participants were included in this study. The specific screening process for participants is illustrated in Figure 1.

The average age of the 13318 participants was 46.5 ± 16.5 years, of which 1012 (7.6%) were diagnosed with chronic diarrhea. Table 1 demonstrates that chronic diarrhea exhibited higher prevalence among older adults (p<0.001), females (p<0.001), those with lower level of education (p<0.001), individuals with reduced PIR (p<0.001), obese participants (p<0.001), hypertensive patients (p<0.001), diabetic patients (p<0.001), and tobacco-exposed individuals (p<0.001).

**Table 1:** Baseline characteristics of participants in a cross-sectional study of tobacco exposure and diarrhea in the NHANES 2005–2010 (N=13318)

*Characteristics*	*All n (%)*	*Non-diarrhea n (%)*	*Diarrhea n (%)*	*p*
**Total**, n	13318	12306	1012	
**Age** (years), mean ± SD	46.5 ± 16.5	46.2 ± 16.5	49.9 ± 15.7	**<0.001**
**Gender**				**<0.001**
Male	6490 (48.7)	6052 (49.2)	428 (42.3)	
Female	6828 (51.3)	6254 (50.8)	584 (57.7)	
**Race**				**0.063**
Mexican American	1037 (7.8)	943 (7.7)	98 (9.6)	
Other Hispanic	544 (4.1)	496 (4.0)	48 (4.7)	
Non-Hispanic White	9594 (72.0)	8899 (72.3)	689 (68.1)	
Non-Hispanic Black	1433 (10.8)	1312 (10.7)	123 (12.2)	
Other	710 (5.3)	656 (5.3)	54 (5.3)	
**Education level**				**<0.001**
Lower than high school	2356 (17.7)	2105 (17.1)	264 (26.0)	
High school	3225 (24.2)	2969 (24.1)	257 (25.4)	
Higher than high school	7737 (58.1)	7232 (58.8)	491 (48.5)	
**Marriage**				**0.002**
Married/cohabitating	8723 (65.5)	8061 (65.5)	661 (65.3)	
Unmarried	2186 (16.4)	2049 (16.6)	133 (13.1)	
Other	2409 (18.1)	2196 (17.8)	218 (21.6)	
**PIR**				**<0.001**
<1.30	2533 (19.0)	2288 (18.6)	254 (25.1)	
1.30–3.49	4787 (35.9)	4429 (36.0)	357 (35.3)	
≥3.50	5999 (45.0)	5590 (45.4)	401 (39.6)	
**BMI**				**<0.001**
Normal weight	4160 (31.2)	3893 (31.6)	259 (25.6)	
Overweight	4472 (33.6)	4158 (33.8)	309 (30.5)	
Obese	4686 (35.2)	4255 (34.6)	444 (43.9)	
**Hypertension**				**<0.001**
Hypertension	4025 (30.2)	3647 (29.6)	390 (38.5)	
Non-hypertension	9293 (69.8)	8659 (70.4)	622 (61.5)	
**Diabetes**				**<0.001**
Diabetes	1048 (7.9)	919 (7.5)	137 (13.5)	
Non-diabetes	12041 (90.4)	11185 (90.9)	846 (83.6)	
Borderline	229 (1.7)	202 (1.6)	29 (2.9)	
**Tobacco exposure**				**<0.001**
Never smokers not exposed to SHS	6658 (50.0)	6218 (50.5)	429 (42.4)	
Never smokers exposed to SHS	383 (2.9)	349 (2.8)	35 (3.4)	
Ex-smokers	3296 (24.7)	3027 (24.6)	272 (26.9)	
Smokers	2981 (22.4)	2712 (22.0)	276 (27.3)	

PIR: poverty-to-income ratio. SHS: secondhand smoke. BMI: body mass index (kg/m2); normal weight <25.0, overweight 25.0–29.9, obese ≥30.0. As this is a descriptive statistic, all variables in the table are independently included in a separate model for calculation. All analyses were conducted using sample weights to ensure national representativeness. The p-value of continuous variables is obtained through linear regression, while the p-value of categorical variables is obtained through chi-squared test.

### The relationship between tobacco exposure and diarrhea

Table 2 shows the logistic regression analyses of different tobacco exposure situations and diarrhea. Compared to never smokers not exposed to SHS, smokers exhibited a high probability of diarrhea. After adjusting for all covariates, the association remained significant (AOR=1.56; 95% CI: 1.25–1.93). However, no significant relationships were observed in either never smokers exposed to SHS (AOR=1.37; 95% CI: 0.87–2.16) or ex-smokers (AOR=1.22; 95% CI: 0.98–1.51) when compared to the never smoker not exposed to SHS.

**Table 2:** Logistic multivariable regression analysis results of the association between different tobacco exposure situations and diarrhea in the NHANES 2005–2010 (N=13318; smokers=2952)

*Characteristics*	*Model 1*		*Model 2*		*Model 3*	
	*OR (95% CI)*	*p*	*AOR (95% CI)*	*p*	*AOR (95% CI)*	*p*
**Tobacco exposure situation**						
Never smokers not exposed to SHS (ref.)	1		1		1	
Never smokers exposed to SHS	1.44 (0.93–2.24)	0.105	1.54 (0.98–2.40)	0.058	1.37 (0.87–2.16)	0.168
Ex-smokers	1.30 (1.06–1.60)	0.011	1.26 (1.01–1.56)	0.038	1.22 (0.98–1.51)	0.079
Smokers	1.48 (1.21–1.81)	<0.001	1.67 (1.15–2.06)	<0.001	1.56 (1.25–1.93)	<0.001
**Smokers only**						
**Smoking time**						
Immediate smokers (ref.)	1		1		1	
Non-immediate smokers	1.62 (1.13–2.30)	0.008	1.63 (1.14–2.34)	0.008	1.69 (1.17–2.45)	0.005
**Smoking duration** (years)						
Continuous (every additional year)	1.02 (1.01–1.03)	0.001	1.02 (1.01–1.03)	0.001	1.02 (1.00–1.03)	0.007
**Smoking dose** (cigarettes per day)						
Continuous (every additional cigarette per day)	1.01 (1.00–1.02)	0.214	1.01 (1.00–1.02)	0.207	1.01 (0.99–1.02)	0.201
≤10 (ref.)	1		1		1	
11–20	0.97 (0.67–1.38)	0.847	0.96 (0.65–1.41)	0.842	0.97 (0.66–1.43)	0.890
>20	1.25 (0.81–1.95)	0.315	1.29 (0.79–2.10)	0.314	1.30 (0.79–2.13)	0.296

Model 1: no covariates were adjusted. Model 2: adjusted for age, gender, and race. Model 3: adjusted as for Model 2 plus education level, marital status, PIR, BMI, hypertension and diabetes. AOR: adjusted odds ratio. SHS: secondhand smoke. The p-value is determined by the Wald test.

**Figure 2:**
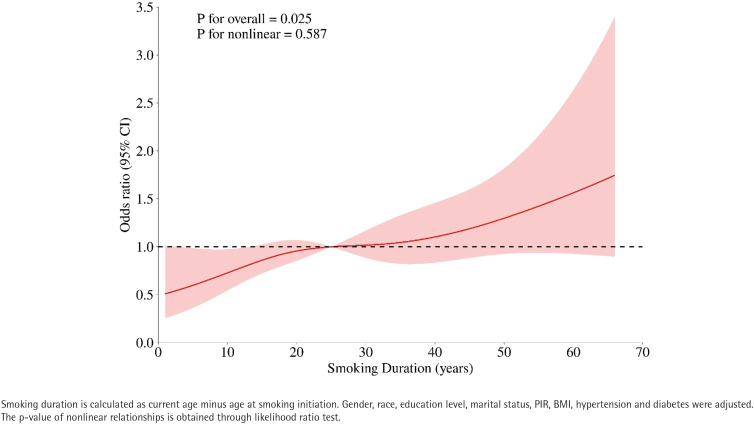
Restricted cubic spline (RCS) analysis of the relationship between smoking duration and diarrhea (only for smokers) in the NHANES 2005–2010 (N=2952)

Among smokers (not including ex-smokers, never smokers not exposed to SHS, never smokers exposed to SHS), those who smoked immediately upon waking exhibited a significantly elevated likelihood of diarrhea after adjusting for all covariates (AOR=1.69; 95% CI: 1.17–2.45) (Table 2). Each additional year of smoking duration was associated with a 2% increase in diarrhea likelihood (AOR=1.02; 95% CI: 1.00–1.03). RCS curve (Figure 2) further confirmed a linear relationship between smoking duration and diarrhea (p for overall=0.025; p for nonlinear=0.587). However, whether smoking dose was treated as a continuous variable (AOR=1.01; 95% CI: 0.99–1.02) or a categorical variable (11–20 vs ≤10 cigarettes per day: AOR=0.97; 95% CI: 0.66–1.43; >20 vs ≤10 cigarettes per day: AOR=1.30; 95% CI: 0.79–2.13), no significant association was observed between smoking dose and diarrhea probability.

Notably, as the analyses investigating the associations of smoking time, smoking duration, and smoking dose with diarrhea were restricted to the smokers, the sample size for the latter three regression models in Table 2 and Figure 2 corresponds to the number of smokers, specifically n=2952. Besides, given the presence of multicollinearity between age and smoking duration, age was not adjusted for as a covariate in Model 2 and Model 3 when examining the relationship between smoking duration and diarrhea (Table 2). And the VIF values of the above two models are both <2.5.

**Figure 3:**
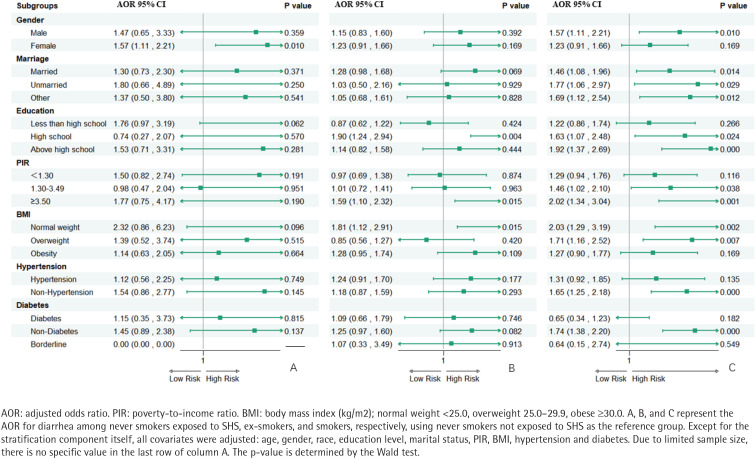
Stratification analysis results on the association between different tobacco exposure situations and diarrhea in the NHANES 2005–2010 (N=13318)

Stratification analysis (Figure 3) confirmed the stability of this association, with more pronounced tobacco exposure-diarrhea relationships observed in males, individuals with higher level of education, those with elevated PIR values, normal weight individuals, non-hypertensive and non-diabetic populations. However, due to the limited sample size, none of the never smokers exposed to SHS with borderline diabetes had diarrhea, so there is no specific value in the last row of column A. Furthermore, it should be clarified that stratified analysis is classified as exploratory analysis without multiple comparison correction and does not provide confirmatory conclusions. Its findings need to be validated in prospective studies with larger sample sizes and thus cannot be used as a basis for stratified intervention in clinical practice for the time being.

### Mediation analysis

In the above study, we have found that compared to never smokers not exposed to SHS, only smokers showed a significant increase in the likelihood of diarrhea, indicating that smokers passed the first step of the Baron and Kenny four-step method for mediation analysis. Therefore, we conducted the follow-up analysis on smokers.

As demonstrated in Table 3, after adjusting for all covariates, smokers exhibited higher odds of exceeding the serum cotinine threshold compared to never smokers not exposed to SHS. However, smokers with elevated serum cotinine levels did not show a statistically significant increase in diarrhea incidence (AOR=1.19; 95% CI: 0.85–1.68).

**Table 3:** Mediating effect analysis of serum cotinine concentration on the association between tobacco exposure and diarrhea in NHANES 2005–2010 (N=3273; smokers=985)

*Characteristics*	*Model 1*		*Model 2*		*Model 3*	
	*OR (95% CI)*	*p*	*AOR (95% CI)*	*p*	*AOR (95% CI)*	*p*
**Tobacco exposure**						
Never smokers not exposed to SHS (ref.)	1		1		1	
Smokers	526.56 (341.68–811.47)	<0.001	719.21 (444.82–1162.86)	<0.001	655.02 (396.55–1081.95)	<0.001
**Cotinine**						
Under threshold (ref.)	1		1		1	
Above threshold	1.12 (0.83–1.52)	0.467	1.34 (0.97–1.84)	0.074	1.19 (0.85–1.68)	0.306

Model 1 no covariates were adjusted. Model 2 adjusted for age, gender, and race. Model 3 adjusted as for Model 2 plus education level, marital status, PIR, BMI, hypertension and diabetes. AOR: adjusted odds ratio. SHS: secondhand smoke. The table presents results of logistic regression analyses conducted separately for the association between tobacco exposure and serum cotinine levels, as well as the relationship between serum cotinine levels and diarrhea. The two regression analyses are all a part of the mediation analysis. The p-value is determined by the Wald test.

Specifically, Table 3 shows the second and third steps of Baron and Kenny’s four-step method for mediation analysis, respectively. As the research results of the third step were not statistically significant, the mediation pathway was not established, and therefore the fourth step analysis was not conducted. Furthermore, it should be clarified that among the 13318 participants, serum cotinine data were available for only 4518 individuals, comprising 2288 never smokers not exposed to SHS and 985 smokers. Consequently, in the first regression analysis investigating the association between tobacco exposure and serum cotinine concentration presented in this table, the sample size was 3273 (n=3273). For the second regression analysis examining the relationship between serum cotinine concentration and diarrhea restricted to smokers, the sample size corresponded to 985 smokers (n=985).

## DISCUSSION

Utilizing pooled data from NHANES 2005–2010, this study represents a systematic investigation of the association between tobacco exposure and diarrhea. We identified that active tobacco exposure was associated with an increased likelihood of diarrhea, while passive exposure and former smoking showed no significant association. Stratification analysis confirmed the robustness of these findings. Both longer smoking duration and smoking immediately after waking were associated with elevated diarrhea likelihood, whereas daily cigarette consumption demonstrated no significant association. Additionally, no mediating effect of serum cotinine was observed in the relationship between tobacco exposure and diarrhea.

As a modifiable lifestyle factor, smoking is one of the earliest identified environmental risk factors for IBD^23^. Previous study^24^ has shown that smokers and ex-smokers exhibit a significantly higher prevalence of CD compared to non-smokers. However, the relationship between smoking and UC remains controversial. A study by Mahid et al.^11^ suggested that former smokers had an increased risk of UC compared to never smokers, while current smokers had a reduced risk. However, a recent Mendelian randomization study^9^ using genetic data from three independent populations identified smoking as a causal risk factor for UC rather than a protective factor. For IBS, a meta-analysis by Nicholas et al.^25^ established smoking as a risk factor for diarrhea-predominant IBS. Although diarrhea is not equivalent to these specific diseases, the above studies have to some extent confirmed a significant association between active tobacco exposure and diarrhea, which is also the main conclusion of our study.

Research on the association between passive tobacco exposure and diarrhea remains limited and inconsistent. A Japanese study^13^ linked passive tobacco exposure to increased UC risk, while van der Heide et al.^14^ identified passive smoking as a risk factor for CD. Conversely, an Israeli multicenter study^15^ found no association between passive smoking and IBD. Additionally, research on passive tobacco exposure and IBS is lacking. In our study, never smokers exposed to SHS showed no statistically significant difference in diarrhea likelihood compared to never smokers not exposed to SHS, suggesting that the association between passive tobacco exposure and diarrhea is not significant. However, due to the limited sample size of never smokers exposed to SHS in this study and the uncertain dose of SHS exposure, we cannot currently draw conclusions about the association between passive tobacco exposure and diarrhea based on this study. Larger scale prospective studies are needed in the future to determine this relationship.

Tobacco smoke is a highly complex aerosol containing over 4000 compounds^26^, and current assessments of tobacco exposure primarily rely on serum cotinine levels, a metabolite of nicotine^27^. Nicotine has been demonstrated to induce multiple intestinal effects, including hypoperfusion of the rectum and of acutely damaged colonic tissue^28^ and reduced smooth muscle tone/contractility^29^. Concurrently, nicotine may modulate the interaction between cytokine profile dysregulation and cell cycle response alterations in UC and CD, thereby influencing disease progression^30^. However, our study revealed no association between serum cotinine concentrations and diarrhea, suggesting other tobacco constituents instead of nicotine may drive this association. Importantly, given IBS substantially higher prevalence compared to UC and CD in diarrheal populations^31,32^, this observation does not negate nicotine’s pathogenic roles in UC and CD pathophysiology, but rather implies nicotine-independent mechanisms may underlie IBS and other diarrhea-predominant intestinal disorders.

Interestingly, our study found that individuals who smoked immediately after waking had a significantly higher probability of developing diarrhea compared to other smokers. Although the mechanism remains unclear, this observation may relate to the fasting state. Reduced intestinal content during fasting may allow tobacco components to directly interact with the gut microbiota and mucosal cells, potentially leading to dysbiosis or oxidative damage^33^. Additionally, fasting-state exposure might enhance the direct stimulation of the vagus nerve and intestinal smooth muscle by tobacco components, triggering contractions and functional diarrhea or IBS^34,35^. Besides, the association between smoking duration and increased diarrhea likelihood may reflect cumulative inflammatory effects over time; this finding also provides novel insights into subsequent mechanistic exploration.

### Strengths and limitations

This study investigated the relationship between different tobacco exposure statuses and diarrhea using a nationally representative adult sample. Additionally, we examined the mediating effect of serum cotinine concentration and discussed the potential mechanisms of action, which provide a theoretical basis for subsequent mechanistic studies and large-scale prospective studies. Furthermore, we adjusted for many potential covariate factors using a broad set of covariates, thereby enhancing the reliability of the study results. However, this study has the following limitations: First, this study is a cross-sectional study; therefore, we cannot infer causality between tobacco exposure and diarrhea, and the results are limited to discussions of association. Second, this study is based on a single NHANES database, so the findings may not be generalizable to other countries, and the extrapolation of the results is limited. Third, although many covariate factors were adjusted for in this study, residual confounding may still exist, which could influence the results. Fourth, a large amount of data in this study was collected based on respondents’ recall, so recall bias and social desirability bias are unavoidable. In addition, this study defined diarrhea using the Bristol Stool Form Scale; although this definition has been recognized in previous studies, according to the World Health Organization, the definition of diarrhea should also consider stool frequency, so the results of this study may have certain biases.

## CONCLUSIONS

Active tobacco exposure demonstrates a significant association with elevated diarrhea likelihood, whereas no substantial associations were observed for passive exposure or ex-smokers, a conclusion reinforced by stratification analysis. Both extended smoking duration and immediate post-awakening smoking were associated with increased diarrhea likelihood, though daily cigarette consumption showed no significant association. Furthermore, serum cotinine levels exhibited no mediating effect in this relationship.

## Data Availability

The data supporting this research are available from the following link: https://wwwn.cdc.gov/nchs/nhanes/Default.aspx.

## References

[1] Li X, Li J, Cao Z, Kang N. The incidence of chronic diarrhea decreases with increasing serum calcium levels: a cross-sectional study based on NHANES 2005-2010. BMC Gastroenterol. 2023;23(1):394. doi:10.1186/s12876-023-03029-2 37968590 PMC10647030

[2] GBD 2016 Diarrhoeal Disease Collaborators. Estimates of the global, regional, and national morbidity, mortality, and aetiologies of diarrhoea in 195 countries: a systematic analysis for the Global Burden of Disease Study 2016. Lancet Infect Dis. 2018;18(11):1211-1228. doi:10.1016/S1473-3099(18)30362-1 30243583 PMC6202444

[3] Sandler RS, Stewart WF, Liberman JN, Ricci JA, Zorich NL. Abdominal pain, bloating, and diarrhea in the United States: prevalence and impact. Dig Dis Sci. 2000;45(6):1166-1171. doi:10.1023/a:1005554103531 10877233

[4] Wang LP, Zhou SX, Wang X, et al. Etiological, epidemiological, and clinical features of acute diarrhea in China. Nat Commun. 2021;12(1):2464. doi:10.1038/s41467-021-22551-z 33927201 PMC8085116

[5] Peng Y, Liu F, Qiao Y, et al. Association of abnormal bowel health with major chronic diseases and risk of mortality. Ann Epidemiol. 2022;75:39-46. doi:10.1016/j.annepidem.2022.09.002 36116757

[6] Lugg ST, Scott A, Parekh D, Naidu B, Thickett DR. Cigarette smoke exposure and alveolar macrophages: mechanisms for lung disease. Thorax. 2022;77(1):94. doi:10.1136/thoraxjnl-2020-216296 33986144 PMC8685655

[7] Parmar MP, Kaur M, Bhavanam S, et al. A systematic review of the effects of smoking on the cardiovascular system and general health. Cureus. 2023;15(4):e38073. doi:10.7759/cureus.38073 37234135 PMC10208588

[8] Somerville KW, Logan RF, Edmond M, Langman MJ. Smoking and Crohn’s disease. Br Med J (Clin Res Ed). 1984;289(6450):954-956. doi:10.1136/bmj.289.6450.954 PMC1443150 6435736

[9] Yuan S, Chen J, Ruan X, et al. Smoking, alcohol consumption, and 24 gastrointestinal diseases: Mendelian randomization analysis. Elife. 2023;12:e84051. doi:10.7554/eLife.84051 36727839 PMC10017103

[10] Bastida G, Beltrán B. Ulcerative colitis in smokers, non-smokers and ex-smokers. World J Gastroenterol. 2011;17(22):2740-2747. doi:10.3748/wjg.v17.i22.2740 21734782 PMC3122262

[11] Mahid SS, Minor KS, Soto RE, Hornung CA, Galandiuk S. Smoking and inflammatory bowel disease: a meta-analysis. Mayo Clin Proc. 2006;81(11):1462-1471. doi:10.4065/81.11.1462 17120402

[12] Seyedian SS, Nokhostin F, Malamir MD. A review of the diagnosis, prevention, and treatment methods of inflammatory bowel disease. J Med Life. 2019;12(2):113-122. doi:10.25122/jml-2018-0075 31406511 PMC6685307

[13] Nishikawa A, Tanaka K, Miyake Y, et al. Active and passive smoking and risk of ulcerative colitis: a case-control study in Japan. J Gastroenterol Hepatol. 2022;37(4):653-659. doi:10.1111/jgh.15745 34845747

[14] van der Heide F, Dijkstra A, Weersma RK, et al. Effects of active and passive smoking on disease course of Crohn’s disease and ulcerative colitis. Inflamm Bowel Dis. 2009;15(8):1199-1207. doi:10.1002/ibd.20884 19170191

[15] Eliakim R, Reif S, Lavy A, et al. Passive smoking in patients with inflammatory bowel disease: an Israeli multicentre case-control study. Eur J Gastroenterol Hepatol. 2000;12(9):975-979. doi:10.1097/00042737-200012090-00002 11007132

[16] Lloyd-Jones DM, Ning H, Labarthe D, et al. Status of cardiovascular health in US adults and children using the American Heart Association’s new “Life’s Essential 8” metrics: prevalence estimates from the National Health and Nutrition Examination Survey (NHANES), 2013 through 2018. Circulation. 2022;146(11):822-835. doi:10.1161/CIRCULATIONAHA.122.060911 35766033

[17] Riegler G, Esposito I. Bristol scale stool form. A still valid help in medical practice and clinical research. Tech Coloproctol. 2001;5(3):163-164. doi:10.1007/s101510100019 11875684

[18] Zhuang Y, Li L, Sun J, Zhang Y, Dai F. Association of body roundness index with chronic diarrhea and constipation, NHANES 2005-2010. J Health Popul Nutr. 2025;44(1):50. doi:10.1186/s41043-025-00793-7 40022226 PMC11869572

[19] Bernert JT Jr, Turner WE, Pirkle JL, et al. Development and validation of sensitive method for determination of serum cotinine in smokers and nonsmokers by liquid chromatography/atmospheric pressure ionization tandem mass spectrometry. Clin Chem. 1997;43(12):2281-1291. 9439445

[20] Benowitz NL, Bernert JT, Caraballo RS, Holiday DB, Wang J. Optimal serum cotinine levels for distinguishing cigarette smokers and nonsmokers within different racial/ethnic groups in the United States between 1999 and 2004. Am J Epidemiol. 2009;169(2):236-248. doi:10.1093/aje/kwn301 19019851

[21] Shi YY, Zheng R, Cai JJ, Qian SZ. The association between triglyceride glucose index and depression: data from NHANES 2005-2018. BMC Psychiatry. 2021;21(1):267. doi:10.1186/s12888-021-03275-2 34030657 PMC8146990

[22] Zheng B, Huang Z, Wang Z, et al. Association of dietary creatine intake from meat protein sources with different types of intestinal problems: insights from NHANES 2005-2010. Front Nutr. 2025;12:1586569. doi:10.3389/fnut.2025.1586569 40786687 PMC12331507

[23] Piovani D, Danese S, Peyrin-Biroulet L, Nikolopoulos GK, Lytras T, Bonovas S. Environmental risk factors for inflammatory bowel diseases: an umbrella review of meta-analyses. Gastroenterology. 2019;157(3):647-659.e4. doi:10.1053/j.gastro.2019.04.016 31014995

[24] Higuchi LM, Khalili H, Chan AT, Richter JM, Bousvaros A, Fuchs CS. A prospective study of cigarette smoking and the risk of inflammatory bowel disease in women. Am J Gastroenterol. 2012;107(9):1399-1406. doi:10.1038/ajg.2012.196 22777340 PMC3667663

[25] Talley NJ, Powell N, Walker MM, et al. Role of smoking in functional dyspepsia and irritable bowel syndrome: three random population-based studies. Aliment Pharmacol Ther. 2021;54(1):32-42. doi:10.1111/apt.16372 33983640

[26] Zuo H, Faiz A, van den Berge M, et al. Cigarette smoke exposure alters phosphodiesterases in human structural lung cells. Am J Physiol Lung Cell Mol Physiol. 2020;318(1):L59-L64. doi:10.1152/ajplung.00319.2019 31664853

[27] Benowitz NL, Bernert JT, Foulds J, et al. Biochemical verification of tobacco use and abstinence: 2019 update. Nicotine Tob Res. 2020;22(7):1086-1097. doi:10.1093/ntr/ntz132 31570931 PMC7882145

[28] Hatoum OA, Binion DG, Otterson MF, Gutterman DD. Acquired microvascular dysfunction in inflammatory bowel disease: Loss of nitric oxide-mediated vasodilation. Gastroenterology. 2003;125(1):58-69. doi:10.1016/s0016-5085(03)00699-1 12851871

[29] Green JT, Richardson C, Marshall RW, et al. Nitric oxide mediates a therapeutic effect of nicotine in ulcerative colitis. Aliment Pharmacol Ther. 2000;14(11):1429-1434. doi:10.1046/j.1365-2036.2000.00847.x 11069313

[30] Aldhous MC, Prescott RJ, Roberts S, Samuel K, Waterfall M, Satsangi J. Does nicotine influence cytokine profile and subsequent cell cycling/apoptotic responses in inflammatory bowel disease? Inflamm Bowel Dis. 2008;14(11):1469-1482. doi:10.1002/ibd.20523 18618634

[31] Kaplan GG, Windsor JW. The four epidemiological stages in the global evolution of inflammatory bowel disease. Nat Rev Gastroenterol Hepatol. 2021;18(1):56-66. doi:10.1038/s41575-020-00360-x 33033392 PMC7542092

[32] Oka P, Parr H, Barberio B, Black CJ, Savarino EV, Ford AC. Global prevalence of irritable bowel syndrome according to Rome III or IV criteria: a systematic review and meta-analysis. Lancet Gastroenterol Hepatol. 2020;5(10):908-917. doi:10.1016/S2468-1253(20)30217-X 32702295

[33] Papoutsopoulou S, Satsangi J, Campbell BJ, Probert CS. Review article: impact of cigarette smoking on intestinal inflammation-direct and indirect mechanisms. Aliment Pharmacol Ther. 2020;51(12):1268-1285. doi:10.1111/apt.15774 32372449

[34] Populin LC, Rajala AZ, Matkowskyj KA, et al. Characterization of idiopathic chronic diarrhea and associated intestinal inflammation and preliminary observations of effects of vagal nerve stimulation in a non-human primate. Neurogastroenterol Motil. 2024;36(9):e14876. doi:10.1111/nmo.14876 39072841 PMC11321913

[35] Johnson JC, Geesala R, Zhang K, Lin YM, M’Koma AE, Shi XZ. Smooth muscle dysfunction in the pre-inflammation site in stenotic Crohn’s-like colitis: implication of mechanical stress in bowel dysfunction in gut inflammation. Front Physiol. 2023;14:1215900. doi:10.3389/fphys.2023.1215900 37520831 PMC10375711

